# Emergence and Persistence of Resistance-Associated Substitutions in HCV GT3 Patients Failing Direct-Acting Antivirals

**DOI:** 10.3389/fphar.2022.894460

**Published:** 2022-04-27

**Authors:** Saima Mushtaq, Asraf Hussain Hashmi, Amjad Khan, Syed Muhammad Asad Raza Kazmi, Sobia Manzoor

**Affiliations:** ^1^ Department of Healthcare Biotechnology, Atta-ur-Rahman School of Applied Biosciences, National University of Sciences and Technology, Islamabad, Pakistan; ^2^ Institute of Biomedical and Genetic Engineering, Islamabad, Pakistan; ^3^ Department of Pharmacy, Quaid-i-Azam University, Islamabad, Pakistan; ^4^ Fauji Foundation Hospital, Rawalpindi, Pakistan

**Keywords:** hepatitis C virus, genotype 3, direct-acting antiviral, relapse, resistance-associated substitutions

## Abstract

**Background:** The hepatitis C virus has a high mutation rate, which results in the emergence of resistance-associated substitutions (RASs). Despite direct-acting antivirals (DAAs) efforts to treat chronically infected HCV genotype 3 (GT3) patients, there are concerns about the emergence and persistence of RASs in DAA failures. The objective of this study was to determine the prevalence of clinically relevant RASs in HCV NS5A and NS5B regions before and after treatment to better understand the role of RASs in treatment failures.

**Methods:** Viral RNA was extracted before and after treatment from serum samples. NS5A and NS5B regions of HCV were amplified by nested PCR, followed by Sanger sequencing. The nucleotide sequences were aligned against HCV GT3 reference sequences, and amino acid substitutions were analyzed using the geno2pheno [hcv] webserver.

**Results:** A total of 76 patients failing DAA therapy were stratified from the cohort of 1388. RASs were detected at the baseline in 15/76 patients and at relapse in 20/76 patients with cirrhosis and previously treated with interferons. The most prevalent NS5A RAS was Y93H found in all treatment-failing patients (14/54 in DCV vs. 6/22 in VEL), followed by A62S/T and A30K. No RASs were identified in NS5B. RASs that were present at the baseline persisted through the 24-week follow-up period and were enriched with emerging RASs during the treatment. The presence of RASs may be one of the causes of treatment failures in 26.3% of patients. Amino acid substitutions were present at the baseline in most of the patients with RASs against NS5A inhibitors. Patients with the baseline Y93H and/or A30K relapse more frequently than patients harboring A62S/T.

**Conclusion:** Treatment-failing patients harbored NS5A RASs, and the most frequent were A30K (5/20), A62S/T (20/20), and Y93H (20/20). Direct resistance testing is recommended for optimizing re-treatment strategies in treatment-failing patients.

## Introduction

Genotype 3 (GT3) of the hepatitis C virus (HCV) is the second most prevalent genotype, accounting for approximately 54 million infections globally, or 30% of all HCV cases (Messina et al., 2015). In relation to other genotypes, GT3 infection results in faster liver fibrosis (Bochud et al., 2009), a high degree of hepatic steatosis (Adinolfi et al., 2001), and an increased risk of hepatocellular cancer (Nkontchou et al., 2011), all of which necessitate urgent therapy. Pakistan is the second largest HCV burden country in the world, with 4.5–8.2% HCV seroprevalence (Iqbal et al., 2014; Umer and Iqbal, 2016). The majority of HCV infections are GT3a (69.1%), followed by GT1 (7.1%), 2 (4.2%), and 4 (2.2%) (Umer and Iqbal, 2016; Khan, 2019).

The advent of direct-acting antivirals (DAAs) has transformed the treatment landscape of hepatitis C. Despite this successful therapeutic intervention, no vaccine is available for HCV. DAA therapeutic regimens have an overall cure rate of more than 90%. Although only a small percentage of efficiently treated patients (5%) fail DAAs treatment, given the global prevalence of HCV, this translates into a substantial absolute number of patients who require retreatment. (Foster, 2016; Sarrazin, 2016). The contributing factors for DAAs treatment failure are patient adherence, suboptimal regimens, and drug resistance. Resistance-associated substitutions (RASs) are frequently detected in DAAs treatment failures (Sorbo et al., 2018). Due to the replication errors in HCV, the high rate of mutations contributes to reduced susceptibility to DAAs. If complete viral replication suppression is not achieved during DAAs treatment, previously existing strains with reduced susceptibility can be selected and result in a virological relapse after therapeutic cessation (Pawlotsky, 2011; Lontok et al., 2015). Furthermore, the presence of NS5A RASs with a high fold resistance, combined with other negative factors such as GT3a, cirrhosis, or previous treatment with non-DAAs, may reduce the efficacy of DAAs. (Lontok et al., 2015; Wyles, 2017). The combination of pan-genotypic NS5A and NS5B inhibitors (daclatasvir; DCV or velpatasvir; VEL and sofosbuvir; SOF) has shown greater therapeutic effectiveness in HCV GT3 infections, and this combination is added to “Hepatitis Control Programs” in Pakistan (Leroy et al., 2016; Wyles, 2017; Cornberg et al., 2019; Mushtaq, 2020a).

Sanger sequencing with a 15–20% cut-off level and NGS (next-generation sequencing) with a 1% cut-off level are used for the detection of the RASs in the viral population. However, a cut-off level of 10–20% for clinical relevance is recommended for detecting RASs within the HCV variants (Pawlotsky, 2016; Wyles, 2017; Pawlotsky et al., 2018).

The presence of RASs has been linked to treatment failures. RASs are found in nearly 100% of patients who experience viral breakthrough, while 50–90% in patients who relapse, depending on the genotype, DAA regimen, and the RAS analysis method (Perales et al., 2015; Sarrazin, 2016). The most commonly detected NS5A RASs in patients with therapeutic failure involved multiple amino acid positions (24, 28, 30, 31, 58, 92, and 93) with high persistence of years. On the other hand, NS5B RAS has a very short half-life due to its adverse effect on viral fitness levels. The impact and prevalence of RASs varied between HCV genotypes (Pawlotsky, 2016; Sarrazin, 2016; Zeuzem et al., 2017). The substitution Y93H is a common RAS seen in DCV-failing patients and can be found in a variety of genotypes (Dietz et al., 2018). There are currently no conclusive studies that indicate the need for baseline RAS testing; the baseline Y93H is thought to have an impact on the SOF/VEL therapy outcome in GT3 patients. Nonetheless, except in cirrhotic patients, the presence of Y93H does not appear to have a substantial impact on the outcome of VEL-based treatment. The American Association for the Study of Liver Diseases (AASLD) recommends testing for resistance to NS5A RASs Y93H in GT3-infected treatment-naive individuals with cirrhosis (Foster et al., 2015; Wyles, 2017).

Despite the fact that baseline RASs have a minor impact on overall DAAs treatment success, many examples have shown lower response rates when RASs are present. Continuing viral replication in the face of suboptimal drug pressure results in the enrichment of pre-existing RASs or the accumulation of new RASs, lowering drug susceptibility or increasing viral replicative fitness. Given that 58 million people worldwide have active HCV infections, even 2–5% treatment failure rates would lead to a large number of patients infected with resistant HCV (Howe et al., 2021).

We have evaluated the impact of baseline RASs on treatment outcomes in GT3-infected patients treated with DAAs. Furthermore, the emergence and persistence of NS5A and NS5B RASs possibly associated with drug resistance were also examined in treatment failures.

## Methods

### Patients and Therapeutic Regimens

**FIGURE 1:**
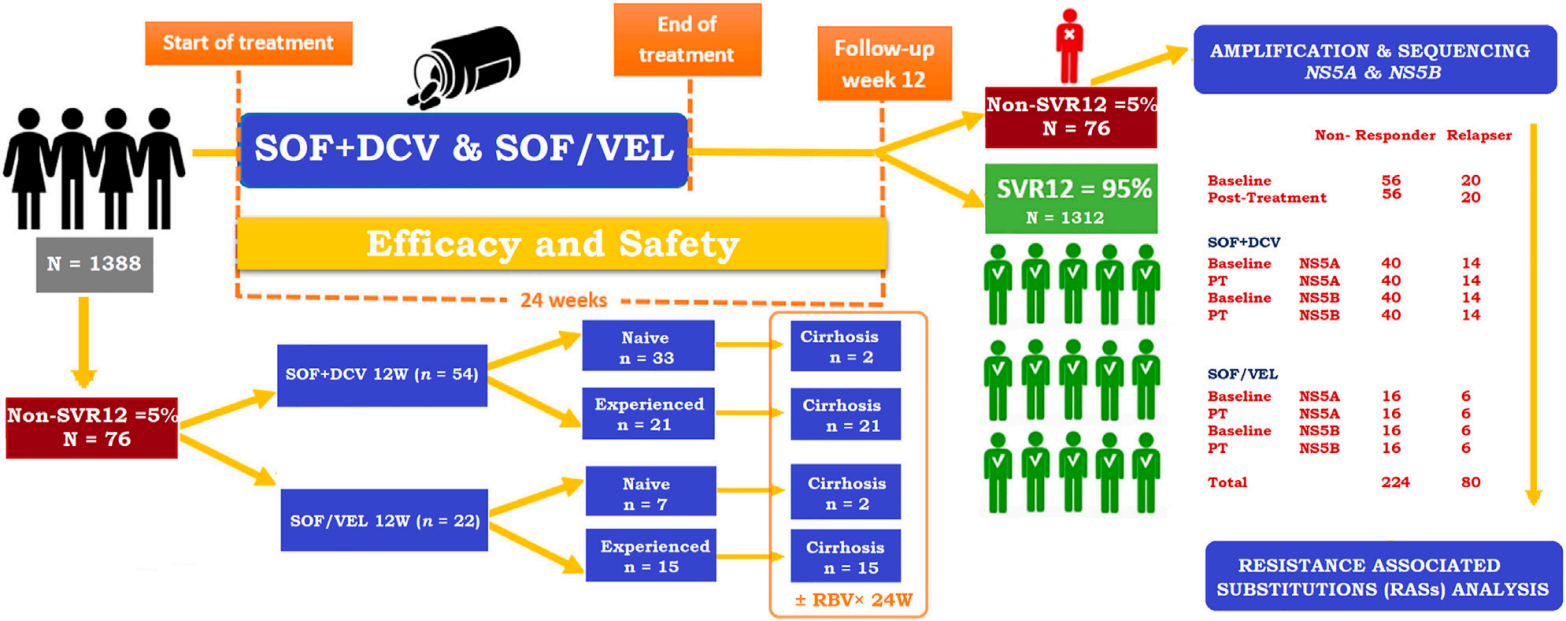
Study flow chart of HCV GT3 patients.

A total of 76 non-SVR HCV GT3 patients were stratified from the cohort of 1388 (January 2019 to January 2020) (Mushtaq, 2020a). HCV RNA was isolated from serum samples of non-SVR patients at the baseline and post-failure in the following two groups of SOF + DCV for 12 weeks in compensated or decompensated cirrhosis and the SOF/VEL group for 12 weeks in compensated or decompensated cirrhosis. Patients were treated with the following DAAs regimens based on the progressive availability of new DAAs and in accordance with national and international recommendations (Figure 1). The inclusion criteria included infection with HCV GT3a and age ≥18 years. Patients who had previously received SOF plus RBV or other DAAs were excluded.

### Study Assessments

The primary outcome was sustained virological response (SVR), defined as undetectable HCV RNA in the serum at end of treatment. Non-SVR patients included non-responders or virological relapse. The virological response to treatment is used to characterize non-response (HCV RNA becomes detectable at treatment completion) or relapse (HCV RNA decreases and remains below the detection limit, but it becomes detectable if treatment is stopped) (Dieterich et al., 2009). The COBAS TaqMan HCV test by Roche Molecular Systems with a lower limit of quantitation (LLOQ) of 25 IU/ml was used to determine HCV-RNA levels at the baseline, on-treatment 4, 8, and 12 weeks and post-treatment 16, 20, and 24 weeks. The HCV genotype, as well as the subtype, was determined using Abbott’s RealTime HCV genotype II assay and confirmed by sequence analysis. Sanger sequencing was used to test for resistance in plasma samples from all patients at the baseline and from patients who had HCV-RNA levels of 1,000 IU/ml in the NS5A and NS5B genes at the end of the treatment. Serum samples of patients were stored at −80°C until the treatment was completed. Adverse event (AE) reporting, clinical laboratory tests, and vital signs were used to assess safety and tolerability.

The primary goal was to find out how common clinically relevant RASs were in DAA treatment-failing patients in both groups of DAAs therapy. Second, the distribution of patients with NS5A and NS5B baseline RASs, the prevalence of people with these baseline NS5A and NS5B RASs who are treatment-failing, and the prevalence of people with these baselines NS5A and NS5B RASs who are not treatment-failing will be determined.

### Laboratory Protocols

Resistance testing of RASs (at baseline and post-treatment) was carried out at the Institute of Biomedical and Genetic Engineering (IBGE), KRL Hospital, Islamabad. The nested PCR method was used to amplify HCV genes from patient samples, which were then sequenced using the Sanger sequencing method.

#### HCV RNA Isolation

The Viral RNA Mini Kit by QIAGEN (Cat. No. 52904) was used to extract all of the RNA. The extracted RNA was also quantified using a NanoDrop 2000 UV-Vis spectrophotometer (Thermo Fisher Scientific). A 260/280 ratio of nearly 1.8 to 2 indicated that the RNA was pure. The RNA was aliquoted as soon as possible after quantification to avoid repeated freeze-thaw cycles.

#### Complementary DNA (cDNA) Synthesis

Complementary DNA was synthesized using Invitrogen’s commercial SuperScriptTM III Reverse Transcriptase (Cat. No. 18080–093) and gene-specific reverse primers. For the synthesis, 50 μL of the reaction volume was used for the final concentration of the reagents. After preparing the reaction mixture, the contents of the tube were gently mixed and incubated for 10 min at 25°C, 55 min at 50°C, and 15 min at 70°C. After incubation, the cDNA was stored at −20°C. The cDNA was used as a template for nested PCR.

#### Primer Design and Nested PCR Optimization

Primers were designed manually using a consensus sequence from the alignment retrieved from the online Los Alamos HCV database. Gradient PCR was used to optimize the primer annealing temperature. Primer sequences for the selected target genes are given in Table 1. Nested PCR was carried out with these primers targeting NS5A and NS5B regions of HCV using Phusion™ High–Fidelity DNA Polymerase by Thermo Fisher Scientific (Cat. No. F530S) Table 2. The amplicons were confirmed by 2% agarose gel electrophoresis by Syngene. Before proceeding to sequencing, PCR-positive samples were purified using a MAGBIO’s HighPrep™ PCR kit (Cat. No. AC-60005).

**TABLE 1:** Primers used for the nested PCR assay.

Primer	ID	Nucleotide sequence of primers (5–3)
*HCVNS5A*	5AEF1	CTC​ATC​GCA​TTC​GCA​TCC​CGG
	5AER1	CAG​GCA​CTT​CCA​CAT​CTC​GTC​CC
	5AIF2	AAC​TGT​CAC​TAG​TCT​GCT​CCG​GCG​GTT​G
	5AIR2	ATG​GAG​GGG​ATC​CGC​GCG​CAA​GGC​GG
*HCVNS5B*	5BEF1	TGA​GCT​AGT​GGA​CGC​CAA​CTT​GTT​ATG
	5BER1	GTT​CTT​CGC​CAT​GAT​GGT​GGT​TGG​AAT
	5BIF2	ACGGAGCGGCTTTACTGC
	5BIR2	CGT​ACC​GCC​CAA​TTA​AAG​AG

**TABLE 2:** Thermal cycler program.

Cycle Step	Nested PCR, step I		Nested PCR, step II^a^		Cycles
	Temp. (°C)	Time	Temp. (°C)	Time	
Initial denaturation	98	30 s	98	30 s	1
Denaturation	98	10 s	98	10 s	
Annealing	66	20 s	64	20 s	35
Extension	72	4 min	72	30 s	
Final extension	72	10 min	72	10 min	1
Hold	4	Hold	4	Hold	Hold

a Nested PCR, step II was performed as step I, with the following changes: 5 ul of the step I product was used as the DNA template, and primers were changed from external to internal. The annealing temperature was changed to 64°C, and the extension time was changed to 30 s.

#### Sanger Sequencing of NS5A and NS5B

After purification of sequencing templates, the BigDye™ Terminator v3.1 Cycle Sequencing Kit by ThermoFisher (Cat. No. 4337454) was used for sequencing reactions. Sequencing reactions were further purified with MAGBIO’s HighPrep™DTR (Cat. No. DT-70005) kit and resuspended in 10 µL of Hi-Di™ Formamide. At both sites, the purified products were run for capillary electrophoresis (Sanger) sequencing on a 3130 Genetic Analyzer (Applied Biosystems™, Thermo Fisher Scientific, USA) with the same primer pair used in the nested PCR.

### Sequence Analysis and Interpretation of RASs

SeqScape^®^ Software version 2.6 by Thermo Fisher Scientific was used for the alignment of forward and reverse nucleotide sequences of all samples with the reference strain of GT3a isolate NZL1 (Accession number D17763.1) to generate a consensus sequence of the HCV quasispecies, which resulted as a mixture of peaks in the electropherogram. The consensus sequences were queried in the web-based mutation detection algorithm, “geno2pheno [hcv]” for the differentiation of the clinically relevant substitutions and their evaluation for potential ramifications. RASs greater than 100X were chosen following the geno2pheno [hcv] rules. Geno2pheno [hcv] (http://hcv.geno2pheno.org) is a key tool for interpreting and assessing HCV sequences for the prediction of resistance against DAAs. (Kalaghatgi et al., 2016). The analysis was performed to look into the RASs in the NS5A and NS5B regions of HCV by comparing scores of 2018 and 2020 EASL guidelines against clinically relevant RASs (Pawlotsky et al., 2018; Pawlotsky, 2020). Furthermore, the RASs reported as affecting DAA treatment outcomes *in vitro* and *in vivo* were also evaluated (Wyles, 2017; Palanisamy et al., 2018; Sorbo et al., 2018). NS5A RASs, i.e., Y93H and A30K, were defined as clinically relevant for HCV GT3 in this study which is also been reported in the literature previously (Hernandez et al., 2013; Wyles, 2017; Di Maio et al., 2018; Dietz et al., 2018; EASL, 2018; Hezode et al., 2018; Ghany et al., 2019; Sharafi et al., 2019). The reference sequence D17763 was used for HCV GT3a. The Sanger sequences of HCV GT3a obtained in this study are available in Supplementary Material. However, they are deposited in the GenBank database under the following accession numbers: ON009333–ON009338.

### Statistics

For data entry and analysis, SPSS (Statistical Package for Social Sciences) software was used. The results of patients’ demographic and laboratory tests were expressed as a number (percentage) for binary variables and as a mean (standard deviation) for continuous variables. The baseline data of the treatment regimens administered (SOF + DCV vs. SOF/VEL) were compared. Student’s t-test was used to compare two groups, and a chi-squared test was used to compare binary variables. The analysis was considered statistically significant when the *p* value was <0.05.

## Results

### Patient Characteristics at the Baseline

In total, 1388 patients with HCV GT3, 972 in the SOF + DCV group and 416 in the SOF/VEL group, were assessed for treatment efficacy previously (Mushtaq, 2020a; Mushtaq, 2020b). Among them, 76/1388 (5%) failed to achieve SVR during DAAs treatment. Detailed demographic and baseline clinical characteristics of the study cohort (n = 76) are described in Table 3. The mean age was 53 years; most patients were females (59%), treatment-experienced (48%), and (53%) cirrhotic. The most common comorbidities at the baseline included diabetes (59%), gastrointestinal disease (89%), and kidney disease (39%). The risk factors correlated with the SVR rate were blood transfusion (92%), surgery (59%), and tobacco smoking (46%). All patients were infected mainly with HCV GT3a. As a result of assignment criteria, more patients treated with SOF/VEL were of old age (58 vs. 51, *p* = 0.04) and cirrhotic (77 vs. 43%, *p* < 0.001). Similarly, most of the patients were INF-experienced in the SOF/VEL group and SOF + DCV group (68 vs. 39%, *p* = 0.02). Significant differences were observed in relapsed patients (26 vs. 27%, *p* = 0.01) of SOF + DCV and SOF/VEL groups, respectively. The significant risk factor associated with the non-SVR was blood transfusion (*p* = 0.03). Similarly, elevated ALT was found to be a significant contributor in both groups of DAAs.

**TABLE 3:** Baseline characteristics and demographics.

	SOF + DCV group (n = 54)	SOF/VEL group (n = 22)	*p* Value
Mean age, yr. (range)	51 (30–75)	58 (35–77)	0.04
Male, n (%)	21 (39)	10 (46)	—
Cirrhosis, n (%)	23 (43)	17 (77)	0.001
Mean HCV RNA, log10 IU/ml (range)	6.2 (3.9–7.1)	6.4 (4.2–7.9)	—
Previous treatment^a^, n (%)	21 (39)	15 (68)	0.02
Non-responder, n (%)	40 (74)	16 (73)	
Relapsers, n (%)	14 (26)	6 (27)	0.01
Significant mutations	—	—	—
Baseline S98G, n (%)	50 (93)	20 (91)	—
Baseline Y93H, n (%)	9 (17)	4 (18)	—
Baseline A62S, n (%)	51 (95)	18 (82)	—
Baseline A30K, n (%)	1 (2)	1 (5)	—
Laboratory data, M±SD	—	—	—
HB (g/dl)	13 ± 2	13 ± 2	—
WBCs (×10^9^/L)	8 ± 2	7 ± 1	—
PLT (×10^9^/L)	203 ± 77	211 ± 80	—
AST (40U/L)	70 ± 34	69 ± 35	—
ALT (40U/L)	80 ± 60	92 ± 70	0.05
Albumin (g/L)	40 ± 3	40 ± 6	—
Creatinine (mg/dl)	99 ± 25	100 ± 26	—
INR	1 ± 0.1	1.1 ± 0.1	—
Comorbidities	—	—	—
Diabetes, n (%)	35 (65)	10 (45)	—
Gastrointestinal disease, n (%)	50 (92)	18 (80)	—
Kidney disease, n (%)	22 (40)	8 (36)	—
Risk factors			
Blood transfusion, n (%)	52 (96)	18 (81)	0.03
Surgery, n (%)	35 (65)	10 (45)	—
Tobacco smoking, n (%)	30 (55)	5 (22)	—

a Pegylated interferon (Peg/INF) plus ribavirin.

Bold values represent statistical significance.

### Baseline Prevalence of NS5A and NS5B Substitutions.

Sequence analysis of 76 non-SVR patients with reference showed several amino acid residue changes in both groups of DAAs at baseline, as shown in Table 4. For NS5A sequences in the DCV + SOF group, the most prevalent substitutions were S14M (92%), A17S (92%), A21T (92%), A62S (94%), S98G (84%), S103P (83%), D126E (80%), F127C (80%), and D172E (92%). The most prevalent substitutions in NS5B sequences of the DCV + SOF group were E258Q (16%), N307G (16%), and A338V (17%). Similarly, the most prevalent substitutions in NS5A sequences of the VEL/SOF group were S14M (92%), A17S (92%), A21T (92%), D172E (90%), H180N (90%), T183V (90%), and N307G (27%) in NS5B. The frequency of natural polymorphisms to respective positions of amino acids in NS5A and NS5B was higher in the SOF + DCV group and then in the SOF/VEL group receiving patients. Most of the clinically relevant RASs were (A62S/T, Y93H, and A30K) in DAAs failing patients. Second, 34.2% of patients with NS5A baseline RASs and 26.3% of people with these baseline NS5A RASs were treatment-failing, and 14.4% of people with these baseline NS5A RASs were not treatment-failing.

**TABLE 4:** Baseline prevalence of amino acid substitutions in NS5A and NS5B.

DCV + SOF group, n (%)				VEL/SOF group, n (%)			
Substitutions	NS5A, 54	Substitutions	NS5B, 54	Substitutions	NS5A, 22	Substitutions	NS5B, 22
R6G	1 (1.8%)	E258Q	9 (16%)	S14M	20 (92.5%)	E258Q	4 (18%)
S14M	50 (92.5%)	N307G	9 (16%)	A17S	20 (92.5%)	N307G	6 (27%)
V15M	2 (3.7%)	A338V	9 (17%)	A21V	20 (92.5%)	A338V	4 (18%)
A17S	50 (92.5%)	Y391C	1 (1.8%)	A30K	1 (5)	—	—
K20R	3 (5.5%)	V490M	1 (1.8%)	L34V	2 (10%)	—	—
A21T	50 (92.5%)	—	—	A62T	4 (18%)	—	—
A25S	5 (9.2%)	—	—	A62S	18 (82)	—	—
I27M	10 (18.5%)	—	—	Y93H	4 (18.2%)	—	—
A30K	1 (1.8%)	—	—	S98G	20 (91%)	—	—
L34V	20 (37%)	—	—	A147P	2 (10%)	—	—
Q40H	10 (18.5%)	—	—	L158I	2 (10%)	—	—
V46G	5 (9.2%)	—	—	R170G	2 (10%)	—	—
W47R	10 (18.5%)	—	—	D172E	19 (90%)	—	—
V52A	6 (11%)	—	—	L179M	7 (31%)	—	—
P58T	4 (7%)	—	—	H180N	19 (90%)	—	—
C59Y	10 (18.5%)	—	—	T183V	19 (90%)	—	—
A62T	18 (33%)	—	—	—	—	—	—
A62S	51 (94.4%)	—	—	—	—	—	—
Y93H	9 (5%)	—	—	—	—	—	—
S98G	50 (93%)	—	—	—	—	—	—
S103P	45 (83%)	—	—	—	—	—	—
P104H	20 (37%)	—	—	—	—	—	—
V113R	20 (37%)	—	—	—	—	—	—
A114S	20 (37%)	—	—	—	—	—	—
N116S	30 (55.5%)	—	—	—	—	—	—
D126E	43 (80%)	—	—	—	—	—	—
F127C	43 (80%)	—	—	—	—	—	—
A147P	31 (58%)	—	—	—	—	—	—
L158I	32 (30%)	—	—	—	—	—	—
P163S	10 (18.5%)	—	—	—	—	—	—
R170G	10 (18.5%)	—	—	—	—	—	—
D172E	50 (92.5%)	—	—	—	—	—	—
I173H	31 (58%)	—	—	—	—	—	—
V177E	10 (18.5%)	—	—	—	—	—	—
M176T	16 (30%)	—	—	—	—	—	—
L179M	16 (30%)	—	—	—	—	—	—
H180N	32 (60%)	—	—	—	—	—	—
T183V	20 (37%)	—	—	—	—	—	—
I184L	10 (18.5%)	—	—	—	—	—	—
G185V	10 (18.5%)	—	—	—	—	—	—
S207A	20 (37%)	—	—	—	—	—	—

### Treatment Characteristics and RAS Prevalence at the Baseline

Among non-SVR patients, 56/76 (74%) patients were non-responders, while 20/76 (26%) patients relapsed after treatment completion. In total, 54 patients from the SOF + DCV group and 22 from the SOF/VEL group were assessed for RAS analyses at the baseline in both genes (*NS5A* and *NS5B*). Among them, 14 patients were found to be relapsers in the SOF + DCV group and six patients from the SOF/VEL group. They were assessed for RAS analyses in both genes (*NS5A* and *NS5B*) at pre- and post-treatment. However, 40 patients were non-responders in the SOF + DCV group and 16 patients from the SOF/VEL group achieved SVR after 24 weeks of follow-up and had low viral load at week 12. But relapse patients had a high viral load at week 12. Thus, week 12 was found to be significant (*p* = 0.05). Regarding follow-up data, relapse patients were consistent in their therapy, and follow-ups and non-responders were inconsistent in their therapy and follow-ups. Simultaneously, 24 weeks of extended treatment and adding RBV were administered to 77% (17/22) in the SOF/VEL group compared to only 43% (23/54) in the SOF + DCV group (Table 5). The prevalence of RASs was relatively high in relapse patients rather than in non-responders at the baseline. Patients with a high prevalence of RASs at the baseline were further checked for RAS analysis at treatment outcome. Surprisingly, patients with baseline RASs were the majority relapsers. The main reason for treatment failure was the enrichment of RASs. The baseline Y93H was found to be a significant contributor toward resistance development in the treatment - failing patients, especially with cirrhosis and a history of IFN-based treatment. The baseline Y93H was found in 75% of patients who relapse the therapy in comparison with non-responders (16%). However, amino acid substitutions (S98G and A62S) were equally high in all non-SVR patients at the baseline.

**TABLE 5:** Treatment characteristics and RAS prevalence in non-responders vs. relapsers at the baseline.

Baseline	Non-responders, n = 54	Relapsers, n = 20	*p* Value
Treatment regimen, n (%)			
Daclatasvir + sofosbuvir	40 (74%)	14 (70%)	—
Velpatasvir/sofosbuvir	16 (30%)	6 (30%)	—
Treatment duration, wk	—	—	
4 weeks	—	3 (15%)	—
8 weeks	—	5 (25%)	—
12 weeks	52 (96%)	20 (100%)	0.05
16 weeks	40 (74%)	15 (75%)	—
20 weeks	14 (26%)	14 (70%)	—
24 weeks	9 (17%)	9 (45%)	—
Addition of ribavirin, n (%)	23 (43%)	17 (77)	—
Cirrhosis n (%)	20 (37%)	20 (100)	—
Previous treatment^a^, n (%)	16 (30%)	18 (90)	—
Significant mutations	—	—	—
Baseline S98G, n (%)	50 (93%)	18 (90%)	—
Baseline Y93H, n (%)	9 (16%)	13 (65%)	—
Baseline A62S/T/V, n (%)	51 (95%)	20 (100%)	—
Baseline A30K, n (%)	—	2 (10%)	—

a Previous treatment referring to pegylated interferon (Peg/INF) + RBV.

Bold values represent statistical significance.

### Effect of Acquired and Persisted RASs in the NS5A Gene

**FIGURE 2:**
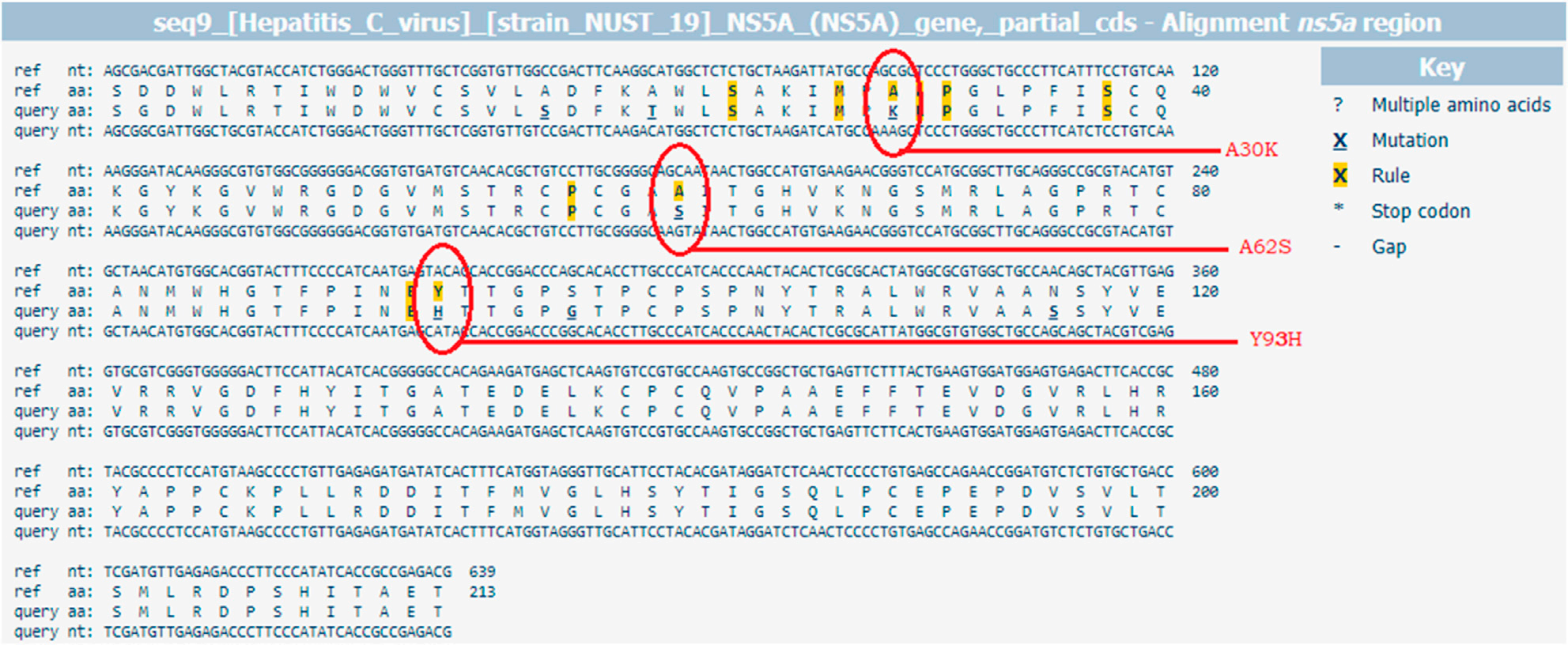
G2P [hcv] web-based interpretation tool for RAS identification through HCV sequence alignment with the reference sequence D17763.

For further analysis of acquired and persistent RASs against NS5A inhibitors, sequences were analyzed by geno2pheno [hcv] 0.92 algorithms (Figure 2) in 20 relapse patients in the DCV + SOF group (n = 14) and DCV + SOF group (n = 6), respectively (Table 6). The results obtained from the analysis of patients’ samples at the baseline revealed that all displayed one substitution on the scored position A62S/T/V (amino acid position conforming resistance) on the *NS5A* gene subjected to the selective pressure of DAA therapy. They will have reduced susceptibility to elbasvir, ledipasvir, and pibrentasvir. Most of the relapsers had RAS Y93H at the baseline which was further maintained and enriched at post-treatment failure.

**TABLE 6:** Distribution of RASs in 20/76 treatment failing patients at pre- and post-treatment.

Patient ID	RASs	NS5A inhibitors				
		Daclatasvir	Elbasvir	Ledipasvir	Pibrentasvir	Velpatasvir
D1 baseline	A62S	RS	S	S	S	S
D1 failure	A62S and Y93H	R	R	R	RS	R
D2 baseline	A62S and A30K	R	R	R	RS	R
D2 failure	A62S, A30K, and Y93H	R	R	R	RS	R
D3 baseline	A62S and Y93H	R	R	R	RS	R
D3 failure	A62S, A30K, and Y93H	R	R	R	RS	R
D4 baseline	A62S and Y93H	R	R	R	RS	R
D4 failure	A62S, A30K, and Y93H	R	R	R	RS	R
D5 baseline	A62T	R	R	R	RS	R
D5 failure	A62T and Y93H	RS	S	S	S	S
D6 baseline	A62S and Y93H	R	R	R	RS	R
D6 failure	A62S and Y93H	R	R	R	RS	R
D7 baseline	A62T and Y93H	R	R	R	RS	R
D7 failure	A62T and Y93H	R	R	R	RS	R
D8 baseline	A62T and Y93H	R	R	R	RS	R
D8 failure	A62T and Y93H	R	R	R	RS	R
D9 baseline	A62S	RS	S	S	S	S
D9 failure	A62S, A30K, and Y93H	R	R	R	RS	R
D10 baseline	A62T and Y93H	R	R	R	RS	R
D10 Failure	A62T, Y93H	R	R	R	RS	R
D11 baseline	A62V	RS	S	S	S	S
D11 failure	A62V and Y93H	R	R	R	RS	R
D12 baseline	A62S and Y93H	R	R	R	RS	R
D12 failure	A62S and Y93H	R	R	R	RS	R
D13 baseline	A62T	RS	S	S	S	S
D13 failure	A62T and Y93H	R	R	R	RS	R
D14 baseline	A62S and Y93H	R	R	R	RS	R
D14 failure	A62S and Y93H	R	R	R	RS	R
V1 baseline	A62S	RS	S	S	S	S
V1 failure	A62S and Y93H	R	R	R	RS	R
V2 baseline	A62S	RS	S	S	S	S
V2 failure	A62S and Y93H	R	R	R	S	R
V3 baseline	Y93H	R	R	R	S	R
V3 failure	Y93H	R	R	R	S	R
V4 baseline	A62S, A30K, and Y93H	R	R	R	RS	R
V4 failure	A62S, A30K, and Y93H	R	R	R	RS	R
V5 baseline	A62T and Y93H	R	R	R	RS	R
V5 failure	A62T and Y93H	R	R	R	RS	R
V6 baseline	A62S and Y93H	R	R	R	RS	R
V6 failure	A62S and Y93H	R	R	R	RS	R

Note: Resistance analysis by using the geno2pheno algorithm. RASs, are represented by red color, and substitutions on scored positions are represented by green color.

Abbreviations: R, resistance; RS, reduced susceptibility; S, susceptible.

All substitutions detected at the baseline on NS5A against DCV and VEL were maintained (A62S/T/V and S98G) in treatment-failing patients. However, most of the patients acquired one or two NS5A RASs: A30K, which confers resistance to daclatasvir, and Y93H, which confers resistance to all inhibitors excluding pibrentasvir. Hence, Y93H has been linked to the failure of NS5A inhibitors. The NS5B RASs that confer resistance to sofosbuvir were not identified and studied. Sequences from all 14 DCV patients and six VEL patients were analyzed for the presence of mutations known to NS5A inhibitor RASs at amino acid positions 28, 30, 31, 32, 58, 92, and 93. Before treatment, DCV patients had NS5A RASs (cut-off 20%) 1/14, 14/14, and 9/14 at positions 30, 62, and 93 of amino acids, and those after treatment were 4/14, 14/14, and 14/14. However, in six VEL patients, before treatment, NS5A RASs were 1/6, 6/6, and 4/6 in DCV patients at positions 30, 62, and 93 of amino acids, and after treatment, they were 1/6, 6/6, and 6/6, respectively. In Table 7, RASs that were present at the baseline persisted through the treatment period and were enriched with emerging RASs during the treatment, i.e., paired RASs (A30K + A62S + Y93H) and (A62T + Y93H) frequency 0–28.6%, 21.4–35.7% pre- and post-DCV treatment, respectively. Similarly, the paired RAS (A62S + Y93H) frequency increased from 16.6 to 50% from pre-to post-VEL treatment, respectively. Most of the paired RASs emerged during the treatment period, indicating that they acquired under drug pressure and lead to treatment failing. All of the RASs present at the pre-treatment stage persisted throughout the treatment and appeared post treatment in the NS5A region of HCV, indicating their long half-life.

**TABLE 7:** Frequency of RASs to DAAs at pre-and post-treatment in the NS5A region of HCV GT3 treatment-failing patients.

RAS/Paired RAS	Drugs			
	Pre-treatment frequency (%)		Post-treatment frequency (%)	
	Daclatasvir	Velpatasvir	Daclatasvir	Velpatasvir
A30K	0	0	0	0
A62S	2/14 (14.2)	2/6 (33.3)	0	0
A62T	2/14 (14.2)	0	0	0
A62V	1/14 (7.1)	0	0	0
Y93H	0	1/6 (16.6)	0	1/6 (16.6)
A30K + A62S	1/14 (7.1)	0	0	0
A30K + Y93H	0	0	0	0
A62V + Y93H	0	0	1/14 (7.1)	0
A62T + Y93H	3/14 (21.4)	1/6 (16.6)	5/14 (35.7)	1/6 (16.6)
A62S + Y93H	5/14 (35.7)	1/6 (16.6)	4/14 (28.6)	3/6 (50)
A30K + A62S + Y93H	0	1/6 (16.6)	4/14 (28.6)	1/6 (16.6)

Safety and tolerability of DAAs among relapsers.

The association between the treated groups and adverse events noted in on-treatment relapse patients during the period of study is shown in Table 8. Both groups of treatment were well-tolerated, with no AEs requiring treatment discontinuation. There were no fatalities reported. A strong association was revealed between adverse events (Skin rashes and oral ulcers) and the treatment group. Patients receiving SOF + DCV had more adverse effects (skin rashes: 50 vs. 45% and oral ulcers: 45 vs. 40%) than those receiving SOF/VEL, respectively.

**TABLE 8:** On-treatment adverse events in relapse patients.

Patients, n (%)	SOF + DCV	SOF/VEL	*p* Value
	Relapsers (n = 14)	Relapsers (n = 6)	
Skin rashes with bruising	7 (50)	3 (45)	0.01
Nausea	3 (22)	1 (17)	—
Edema	6 (40)	2 (35)	—
Weight loss	3 (22)	1 (17)	—
Fatigue	10 (71)	3 (50)	—
Oral ulcers and bleeding	6 (45)	3 (40)	0.05

Bold values represent statistical significance.

In addition, these abnormalities were not caused directly by the treatment regimens but were clinically linked to the fibrosis stage of the liver and its elevated enzymes as these patients were cirrhotic by nature.

## Discussion

The concept of viral resistance is the selection of viral variants that permit the substitution of an amino acid in the viral therapeutic targets making the virus less susceptible to the inhibitory effect of the drug (Pawlotsky, 2011). HCV resistance to DAAs is driven by the selection of mutations in the targeted genes (*NS3*, *NS5A*, and *NS5B*) under the drug (DAAs) pressure (Pawlotsky, 2016). RASs are the amino acid substitutions that confer resistance, and the viral variants that carry these RASs having reduced susceptibility to the DAAs are called resistant variants (RV) (Jiménez-Pérez et al., 2016). The RAS is defined by the type of HCV (genotype and subtype), the position of amino acid, and protein of HCV *via* international recommendations (EASL, 2018). The emergence and type of RASs are determined by the genotype and the drug to which it was exposed (Dietz et al., 2018). Furthermore, it can arise from DAA exposure and exist naturally in naive patients with HCV. RASs seem to be more prevalent in GT1a and GT3a than in other genotypes (Baumert et al., 2019).

Presently, DAA-based regimens cure most HCV patients. Despite this, virological failure can result in 2–5% of patients, especially in accordance with the development of RASs. The presence of RASs in DAA-failing patients may jeopardize the efficacy of second-line treatment and hence is a major priority for successful re-treatment (Lontok et al., 2015).

This study aimed to illustrate the clinically relevant RASs in NS5A and NS5B (DAAs-targeted genes) at pre-and post-treatment among HCV GT3 patients for understanding the role of RASs in the failure of the treatment. When we began this real-world study, we hypothesized that the clinically relating RASs in GT3 should be Y93H. This is because *in vitro* data from the literature show that Y93H confers a high degree of resistance to DCV and VEL, with resistance fold-change values of 2100 and 700, respectively, when compared to GT3a wildtype replicons (Hernandez et al., 2013; Lawitz, 2016). The clinical trials of ALLY and ASTRAL proved that the presence of baseline Y93H in GT3 cirrhotic patients is associated with lower SVR rates to treatment with SOF + DCV and SOF/VEL (Foster, 2016; Goujon et al., 2020). Similarly, our findings are in alliance with these findings where baseline Y93H in GT3 cirrhotic patients (22/76) taking SOF + DCV and SOF/VEL lead to lower SVR rates.

According to the EASL guidelines, baseline RAS testing of Y93H in GT3 patients before starting the SOF/VEL treatment is recommended. The findings of the baseline Y93H will include RBV with/without extension of treatment duration. However, these guidelines were for retreatment against NS5A failures from 2014–2017 for GT3 patients (Liver, 20182018). Therefore, it was important for the study groups to evaluate treatment outcomes based on the Y93H baseline analysis. As shown in Table 5, 13/22 patients in both groups with baseline Y93H were DAA-failing patients. As a result, it appeared that Y93H had a negative impact on treatment outcomes. At the time of relapse, the Y93H RAS in the NS5A region was maintained and enriched with multiple mutations, including S98G and A62S/T/V. In some HCV GT3 patients with baseline Y93H, an emergent S62L and S98G substitution have been identified at treatment failure (Dietz et al., 2018). Their presence at pre-treatment can affect post-treatment sustained virological response (SVR) to DCV-based therapy (Lontok et al., 2015; Pawlotsky, 2016; Zeuzem et al., 2017; Sorbo et al., 2018).

Cirrhosis is an advance and critical condition of chronic HCV infection because it indicates a long-term viral infection accompanied by vigorous viral replication, which may result in viral fitness (Sarrazin, 2016; Smith et al., 2019). In this study, RASs were found in 20/76 DAA-failing patients, 18 of whom were cirrhotic and had previously received IFN-based treatment, and RASs Y93H were found in 20 of the cirrhotic patients. Our findings are consistent with previous research that amino acid substitutions/RASs, particularly clinically relevant RASs, are more common in patients with cirrhosis than in non-cirrhosis patients (Sarrazin, 2016; Smith et al., 2019).

The RASs against NS5A inhibitors result in treatment failure and are regarded as a major threat to HCV treatment and eradication (Zeuzem et al., 2017). In terms of behavior, NS5A RASs can cause a resistance fold-change of more than two, and those that cause a resistance fold-change of more than 100 are known as RASs >100X (Sharafi et al., 2019) For example, substitution in codon (93) can change the related amino acid in the NS5A protein from Y to H, N, and C (Y93H/N/C) and lead to resistance in many NS5A inhibitors (Issur and Götte, 2014). Nonetheless, the viral genotype/subtype is considered in determining the fold-change (Hezode et al., 2018; Liver, 20182018; Sharafi et al., 2019). In this research, baseline RASs were investigated in 76 HCV GT3a patients, the most widespread GT in Pakistan, and regarded as the “difficult-to-treat genotype” (Iqbal et al., 2014; Messina et al., 2015; Umer and Iqbal, 2016). Herein, most of the identified substitutions Y93H paired with RASs (A30K + A62S + Y93H) 0–28.6% pre-and post-DCV treatment, respectively. Similarly, the paired RAS (A62S + Y93H) frequency increased from 16.6 to 50% from pre- to post VEL treatment, respectively. According to some studies, RASs in aa 93 (Y93H) are considered a >100 resistance fold-change NS5A RAS, especially when they appeared as a paired substitution with RASs in aa 30 in HCV 3a (Kjellin et al., 2019; Smith et al., 2019). Viral populations having one or more RASs have fitness levels closer to the wild-type virus and may accumulate through the selection pressure or emerge during the suboptimal conditions of the treatment (Pawlotsky, 2016). NS5A RAS has been shown to persist for at least 1-year post-treatment failure and may impact retreatment with some NS5A inhibitor-containing regimens (McPhee, 2014). So the identification of RASs may guide the choice of the most appropriate drugs for HCV retreatment. In our case, 19/20 patients with paired RASs were treatment-failing against NS5A inhibitors, so second-line therapy has to be different than the present.

In our study, there could be various confounding factors in terms of outcome in the DAA group. The significant negative predictors described in (Table 3) for treatment outcomes were found in the SOF/VEL group compared to those of the SOF + DCV group in terms of the proportion of treatment-experienced patients (68 vs. 39%), older age (58 vs. 51%), and patients with cirrhosis (77 vs. 43%). However, the SOF + DCV group had a higher rate of comorbidities and risk factors. In addition, the SOF/VEL use was less relative to SOF + DCV due to the latest DAA regimen. It has been shown in a cohort of 2824 GT3 patients that SVR rates were similar in both regimens of SOF + DCV or SOF/VEL (Belperio, 2019). However, many studies proved that SOF/VEL has greater efficacy (Sulkowski et al., 2014; Foster et al., 2015; Nelson et al., 2015; Wyles, 2017; Hezode et al., 2018; Liver, 2018). The present study resulted that both regimens SOF/VEL and SOF + DCV were less effective among cirrhotic patients in terms of RAS presence than the non-cirrhotic patients. However, in our previous studies, both regimens were equally effective in the treatment of naïve HCV patients (Mushtaq, 2020a; Mushtaq, 2020b).

Even though the likelihood of achieving SVR with these new DAAs has been increased exponentially in patients with chronic HCV infection who have previously failed HCV antiviral therapy, RASs can appear either before or after treatment with DAAs (Hernandez et al., 2013). Our findings are in alliance with these findings in terms of the presence and prevalence of RASs at the baseline in previously treated GT3 patients—30% in DCV and 90% in VEL group, respectively.

The sequencing techniques have a significant impact on the detection rate of RASs (Pawlotsky, 2020). In this study, we were unable to perform deep sequencing or NSS due to budget constraints. Despite this constraint, HCV GT3 NS5A and NS5B regions were successfully amplified, and Sanger sequencing of 224 baselines and 80 post-treatment PCR products yielded accurate and reliable detection of amino acid substitutions/RASs. Many investigators suggested that if a 15% cut-off is used for the determination of RASs by NGS, both methods can be considered equivalent. Since recent studies have shown that NGS results at a 1% level of sensitivity frequently contribute to the identification of extra RASs that are not associated with clinical failure.(Ghany et al., 2019; Chen et al., 2020), we have used Sanger sequencing for RAS identification in NS5A and NS5B regions of HCV. The NS5A RASs were found in 26.3% of patients. Moreover, many studies have shown the 10–50% baseline range for the overall proportion of NS5A RASs (Issur and Götte, 2014). As a result, the detection rate of NS5A RASs in this study is consistent with the following studies (Wyles, 2017; Dietz et al., 2018; Hernandez et al., 2013; Di Maio et al., 2018; Ghany et al., 2019; Hezode et al., 2018; Sharafi et al., 2019). Similarly, the overall proportion of amino acid substitutions at the baseline in the NS5A and NS5B regions of HCV (Table 4) was very high, which is consistent with prior studies (Lontok et al., 2015; Sarrazin, 2016; Gane et al., 2017; Palanisamy et al., 2018; Rahimi, 2021).

In terms of baseline RASs, the HCV genotype and subtype are among the most influential parameters. In addition, various studies have found an increased prevalence of NS5A amino acid substitutions in HCV GT3a infection, and in this study, the prevalences of NS5A amino acid substitutions were 100X (S14M (92%), A17S (92%), A21T (92%), A62S (94%), S98G (84%), S103P (83%), D126E (80%), F127C (80%), and D172E (92%) in GT3 HCV patients, which were consistent with previous findings (Sarrazin, 2016; Smith et al., 2019; Pawlotsky, 2020) and indicating HCV heterogeneity.

Sofosbuvir is a potent inhibitor against NS5B with a pan-genotypic effect on HCV that does not cause viral resistance in GT1 and 3, with cirrhosis, and a history of previous treatment. Notwithstanding being mutation prone, SOF-resistant variants may not be established or even selectable and correlated with our findings in terms of no RAS in the NS5B region of HCV (Sofia et al., 2010; Gane et al., 2017; Sorbo et al., 2018).

Similarly, both DAA regimens were well-tolerated, with no adverse events leading to discontinuation of treatment. Skin rashes with bruising and oral ulcers were high in patients taking DCV and then VEL-based regimens, respectively. Moreover, these abnormalities were not treatment-emergent but clinically related to liver-related parameters, including ALT and AST, and total bilirubin. These findings are in agreement with the results of the following studies (Hill et al., 2016; Mushtaq, 2020a; Mushtaq, 2020b).

Our findings suggest that in cirrhotic and previously treated HCV GT3 patients, a resistance profiling test should be performed before the start of the therapy. Moreover, our findings are useful, particularly in low- and middle-income countries where generic HCV treatment is becoming more widely available against a variety of genotypes (EASL, 2018; Mushtaq, 2020a).

## Conclusion

To summarize the study’s findings, we observed the important RASs in HCV patients of GT3 from Pakistan. A62S/T, A30K, and Y93H were the most common RASs against NS5A inhibitors, which indicate an increased resistance to some DAA regimens used to treat HCV. While RASs are still a common cause of DAAs failure, identifying them is crucial for optimizing re-treatment strategies. In this rapidly evolving field, stakeholders must work together to assess real-world treatment failure rates and re-treatment success rates in order to inform the field as quickly as possible about regimen selection and the potential need for baseline resistance testing in cirrhotic patients of GT3.

## Study Limitations

We were unable to perform NGS-based sequencing, which can further highlight minority variant frequencies associated with DAAs failure. Second, genotype diversity is lacking as we worked only on GT3. Bioinformatics analysis could reveal all attributes of both proteins, which could be useful for further research into HCV drug resistance and the development of vaccines against HCV. Moreover, cell culture studies can further confirm these pattern of RASs in GT3.

## Data Availability

The datasets presented in this study can be found in online repositories. The names of the repository/repositories and accession number(s) can be found at: https://www.ncbi.nlm.nih.gov; ON009333-ON009338.
