# Supplementary material for: Emergence and Persistence of Resistance-Associated Substitutions in HCV GT3 Patients Failing Direct-Acting Antivirals
Source: Front Pharmacol. 2022 Apr 27;13:894460. doi: 10.3389/fphar.2022.894460 (PMC9091354; doi:10.3389/fphar.2022.894460)
Supplement: Supplementary file 1 [file DataSheet1.docx]

NS5A Gene sequence

>ON009333

AGCGGCGATTGGCTGCGTACCATCTGGGACTGGGTTTGCGCGATGTTGTCCGACTTCAAGACATGGCTCTCTGCTAAGATCATGCCAGCGCTCCCCGGACTGCCCTTTATCTCCTGTCAAAAGGGGTACAAGGGCGTGTGGCGGGGGGACGGCGTGATGTCAACACGCTGTCCTTGCGGGGCATCGATAACTGGCCATGTGAAGAATGGGTCCATGCGGCTTGCAGGGCCGCGTACATGTGCTAACATGTGGCACGGTACTTTCCCCATCAATGAGCATACCACCGGACCCGGCACACCTTGCCCATCACACAATTACACTCGCGCACTATGGCGTGTGTCTGCCAGCAGCTACGTCGAGGTGCGTCGGGTGGGAGACTTCCATTACATCACGGGGGCCACAGAAGATGAGCTCAAGTGTCCGTGCCAAGTGCCGGCTCCTGAGTTCTTCACTGAAGTGGATGGGGTGAGACTCCACCGTTACGCCCCTCCGTGTAAGCCCCTGTTGAGAGATGAGATCACTTTCATGGTAGGGTTACATTCCTACCCGATAGGATCTCAACTCCCCTGTGAGCCCGAACCAGATGTTTCTGTGCTGACCTCGATGTTGAGAGACCCTTCCCATATCACCGCCGAGACG

>ON009334

AGCGGCGATTGGCTGCGTACCATCTGGGACTGGGTTTGCATGGTGTTGTCCGACTTCAAGACATGGCTCTCTGCCAAGATCATGCCCGCGCTCCCTGGGCTGCCATTCATTTCCTGTCAGAAGGGGTACAAGGGCGTGTGGCGGGGGGACGGTGCGATGTCAACACGCTGCCCTTGCGGAGCGTCAATAACCGGTCATGTGAAGAATGGGTCCATGCGGCTTGCAGGGCCGCGTACATGTGCTAACATGTGGCACGGTACTTTCCCCATTAATGAGCACACTACCGGACCCAGCACACCTTGTCCACCACCTAACTACACCCGCGCACTATGGCGCGTGGCTGCCAACAGCTACGTTGAAGTGCGCCGGGTAGGGGACTTCCATTACATTACGGGGGCCACAGAAGATGAGCTCAAGTGTCCATGTCAGGTGCCGGCTCCTGAGTTCTTTACTGAAGTGGACGGAGTGAGACTCCACCGTTATGCTCCTCCATGTAAGCCCCTGTTGAGAGATGATATCACTTTCATGGAAGGGTTGAACTCCTACGCGATAGGATCTCAACTCCCCTGTGAGCCAGAACCGGATGTCTCTGTGCTGACCTCGATGTTGAGAGACCCTTCCCATATCACCGCCGAGACG

>ON009335

AGCGACGATTGGCTACGTACCATCTGGGACTGGGTTTGCTCGGTGTTGGCCGACTTCAAGGCATGGCTCTCTGCTAAGATTATGCCAGCGCTCCCTGGGCTGCCCTTCATTTCCTGTCAAAAGGGATACAAGGGCGGGCGGCGGGGGGATGGTGTAATGTCCACACGCTGTCCTTGCGGGGCAGTGATAACCGGTCATGTGAAGAATGGGTCCATGCGGCTTGCAGGGCCGCGTACATGTGCTAACATGTGGCACGGTACTTTCCCCATCAATGAGCACACCACTGGACCCGGCACACCTTGCCCATCACCCAACTACACTCGCGCACTATGGCGCGTGGCTGCCAACAGCTACGTCGAGGTGCGCCGGGTGGGGGAATGCCATTACATCACGGGGGCCACAGAAGATGAGCTCAAGTGTCCGTGCCAAGTGCCGGCTGCTGAGTTCTTTACTGAAGTAGACGGGGTGAGACTCCACCGTTACGCCCCTCCATGTAAGCCCCTGTTGAGAGATGAGATCACTTTCATGGTAGGGTTGAACTCCTATACGATAGGATCTCAACTCCCCTGTGAGCCAGAACCGGATGTCTCTGTGCTGACCTCGATGTTGAGAGACCCTTCCCATATCACCGCCGAGACG

>ON009336

AGCGGCGATTGGCTGCGTACCATCTGGGACTGGGTTTGCTCGGTGTTGTCCGACTTCAAGACATGGCTCTCTGCTAAGATCATGCCAAAGCTCCCTGGGCTGCCCTTCATCTCCTGTCAAAAGGGATACAAGGGCGTGTGGCGGGGGGACGGTGTGATGTCAACACGCTGTCCTTGCGGGGCAAGTATAACTGGCCATGTGAAGAACGGGTCCATGCGGCTTGCAGGGCCGCGTACATGTGCTAACATGTGGCACGGTACTTTCCCCATCAATGAGCACACCACCGGACCCGGCACACCTTGCCCATCACCCAACTACACTCGCGCATTATGGCGTGTGGCTGCCAGCAGCTACGTCGAGGTGCGTCGGGTGGGGGACTTCCATTACATCACGGGGGCCACAGAAGATGAGCTCAAGTGTCCGTGCCAAGTGCCGGCTGCTGAGTTCTTCACTGAAGTGGATGGAGTGAGACTTCACCGCTACGCCCCTCCATGTAAGCCCCTGTTGAGAGATGATATCACTTTCATGGTAGGGTTGCATTCCTACACGATAGGATCTCAACTCCCCTGTGAGCCAGAACCGGATGTCTCTGTGCTGACCTCGATGTTGAGAGACCCTTCCCATATCACCGCCGAGACG

>ON009337

AGCGGCGATTGGCTGCGTACCATCTGGGACTGGGTTTGCACGGTGTTGTCCGACTTCAAGACATGGCTCTCTGCTAAGATCATGCCCGCACTCCCTGGGTTGCCCTTCATTTCCTGTCAAAAGGGATATAAGGGCGTGTGGCGGGGGGACGGTGTGATGTCTACACGCTGTCCTTGTGGGGCAACAATAACTGGCCATGTGAAGAATGGGTCTATGCGGCTTGCAGGGCCGCGTACGTGTGCTAATATGTGGCACGGTACCTTTCCCATCAATGAGCATACTACTGGACCCAGCACACCTTGCCCATCACCCAATTACACTCGCGCGCTATGGCGAGTGGCTGCCAACAGCTATGTTGAGGTGCGCCGGGTGGGGGACTTCCACTACATCACGGGGGCCACAGAAGATGATCTCAAGCGTCCGTGCCAAGTGCCGGCAGCTGAGTTCTTTACTGAGGTGGATGGAGTGAGACTTCACCGCTACGTCCCTCCATGTAAGCCCCTGTTGAGAGATGATATCTCTTTCATGTTAGGGTTGCATTCCTACACCATAGGATCTCAACTCCCCTCTGAGCCAGAACCTGATGTCTCTGTGCTGCCCTCGATGTTGAGGGACCCCTCCCATATCACCGCCGAGACG

>ON009338

AGCGACGATTGGCTACGTACCATCTGGGACTGGGTTTGCTCGGTGTTGGCCGACTTCAAGGCATGGCTCTCTGCTAAGATTATGCCAGCGCTCCCTGGGGTGCCCTTCATTTCCTGTCAAAAGGGATACAAGGGCGTGTGGCGAGGAGACGGTGTGATGTCAACACGCTGTCCATGCGGAGCGACAATAACCGGCCATGTGAAGAACGGGTCCATGCGGCTTGCAGGACCACGTACATGTGCTAACATGTGGCATGGTACTTTCCCCATCAATGAGCACACCACCGGACCCAGCACACCTTGCCCACCACCTAACTACACTCGTGCACTATGGCGCGTGGCTGCCAACAGCTACGTCGAGGTGCGTCGGGTGGGAGACTTCCATTACATTACGGGGGCCACAGAAGATGAGCTCAAGTGTCCGTGCCAAGTGCCGGCTGCTGAGTTCTTTACTGAAGTGGATGGAGTGAGAATCCACCGTTACGCTCCTCCATGCAAGCCCCTGTTGAGGGATGAGATCACTTTCACAGTAGGGATGAATTCTTACGTGATAGGATCTCAACTCCCTTGTGAGCCAGAACCGGATGTGTCTGTGCTGACCTCGATGTTGAGAGACCCTGCCCATATCACCGCTGAGACG

NS5A Protein sequence

>ON009333

SGDWLRTIWDWVCAMLSDFKTWLSAKIMPALPGLPFISCQKGYKGVWRGDGVMSTRCPCGASITGHVKNGSMRLAGPRTCANMWHGTFPINEHTTGPGTPCPSHNYTRALWRVSASSYVEVRRVGDFHYITGATEDELKCPCQVPAPEFFTEVDGVRLHRYAPPCKPLLRDEITFMVGLHSYPIGSQLPCEPEPDVSVLTSMLRDPSHITAET

>ON009334

SGDWLRTIWDWVCMVLSDFKTWLSAKIMPALPGLPFISCQKGYKGVWRGDGAMSTRCPCGASITGHVKNGSMRLAGPRTCANMWHGTFPINEHTTGPSTPCPPPNYTRALWRVAANSYVEVRRVGDFHYITGATEDELKCPCQVPAPEFFTEVDGVRLHRYAPPCKPLLRDDITFMEGLNSYAIGSQLPCEPEPDVSVLTSMLRDPSHITAET

>ON009335

SDDWLRTIWDWVCSVLADFKAWLSAKIMPALPGLPFISCQKGYKGGRRGDGVMSTRCPCGAVITGHVKNGSMRLAGPRTCANMWHGTFPINEHTTGPGTPCPSPNYTRALWRVAANSYVEVRRVGECHYITGATEDELKCPCQVPAAEFFTEVDGVRLHRYAPPCKPLLRDEITFMVGLNSYTIGSQLPCEPEPDVSVLTSMLRDPSHITAET

>ON009336

SGDWLRTIWDWVCSVLSDFKTWLSAKIMPKLPGLPFISCQKGYKGVWRGDGVMSTRCPCGASITGHVKNGSMRLAGPRTCANMWHGTFPINEHTTGPGTPCPSPNYTRALWRVAASSYVEVRRVGDFHYITGATEDELKCPCQVPAAEFFTEVDGVRLHRYAPPCKPLLRDDITFMVGLHSYTIGSQLPCEPEPDVSVLTSMLRDPSHITAET

>ON009337

SGDWLRTIWDWVCTVLSDFKTWLSAKIMPALPGLPFISCQKGYKGVWRGDGVMSTRCPCGATITGHVKNGSMRLAGPRTCANMWHGTFPINEHTTGPSTPCPSPNYTRALWRVAANSYVEVRRVGDFHYITGATEDDLKRPCQVPAAEFFTEVDGVRLHRYVPPCKPLLRDDISFMLGLHSYTIGSQLPSEPEPDVSVLPSMLRDPSHITAET

>ON009338

SDDWLRTIWDWVCSVLADFKAWLSAKIMPALPGVPFISCQKGYKGVWRGDGVMSTRCPCGATITGHVKNGSMRLAGPRTCANMWHGTFPINEHTTGPSTPCPPPNYTRALWRVAANSYVEVRRVGDFHYITGATEDELKCPCQVPAAEFFTEVDGVRIHRYAPPCKPLLRDEITFTVGMNSYVIGSQLPCEPEPDVSVLTSMLRDPAHITAET
